# Cumulative survival in early-onset unilateral and bilateral breast cancer: an analysis of 1907 Taiwanese women

**DOI:** 10.1038/sj.bjc.6604898

**Published:** 2009-02-03

**Authors:** W-H Kuo, A M-F Yen, P-H Lee, K-M Chen, J Wang, K-J Chang, T H-H Chen, H-S Tsau

**Affiliations:** 1 Department of Surgery, College of Medicine, National Taiwan University Hospital, No. 7, Jhongshan S. Rd., Jhongjheng District, Taipei City 100, Taiwan; 2 Division of Biostatistics, Graduate Institute of Epidemiology, College of Public Health, National Taiwan University, Room 521, No.17, Hsuchow Rd., Jhongjheng District, Taipei City 100, Taiwan; 3 Department of Radiology, College of Medicine, National Taiwan University Hospital, No. 7, Jhongshan S. Rd., Jhongjheng District, Taipei City 100, Taiwan; 4 Angiogenesis Research Center, National Taiwan University, Room 8-46, Floor 8, No. 7, Jhongshan S. Rd., Jhongjheng District, Taipei City 100, Taiwan; 5 Centre of Biostatistics Consultation, College of Public Health, National Taiwan University, Room 540, No.17, Hsuchow Rd., Jhongjheng District, Taipei City 100, Taiwan

**Keywords:** bilateral breast cancer, metachronous breast cancer, survival, synchronous breast cancer, early-onset breast cancer

## Abstract

As the epidemiological pattern of breast cancer in modernising Asian countries differs greatly from that in Western countries, it is worthwhile to investigate the long-term prognoses of unilateral and bilateral breast cancer in these nations. A retrospective cohort study composed of 1907 Taiwanese women was conducted to follow 1863 unilateral and 44 bilateral cases of breast cancer. Time-dependent Cox regression was used to assess the risk of breast cancer death by considering the time course of unilateral and bilateral tumour development. The 15-year survival rates were 68.37, 62.63, and 26.42% for unilateral, synchronous bilateral, and metachronous bilateral breast cancer, respectively. Differences among types were most apparent after 5 years of follow-up. After adjusting for significant prognostic factors, the risk of death for overall bilateral breast cancer was 2.50-fold greater (95% CI, 1.43–4.37) compared to unilateral breast cancer. The corresponding figures were 1.12-fold (95% CI, 0.42–3.02) and 6.11-fold (95% CI, 3.14–11.89) for synchronous and metachronous bilateral breast cancer, respectively. Taiwanese women, who are frequently diagnosed with breast cancer before 50 years of age, showed poorer survival for metachronous bilateral than for synchronous bilateral or unilateral breast cancer. Survival was markedly poorer compared to recent data from Sweden.

Rising breast cancer incidence has been consistently reported in most modernising Asian countries, including Japan, Singapore, Hong Kong, and Taiwan (Shen *et al*, 2005; Sim *et al*, 2006; Matsuno *et al*, 2007; Leung *et al*, 2008), even though organised mass screening programmes, which typically lead to increased reporting of unilateral or bilateral cancer, and adjuvant systematic treatment, which may lead to the reduction of bilateral breast cancer, have not been widely implemented. Moreover, in contrast to the pattern more typically observed in Western countries, breast cancer in modernising Asian countries occurs predominately in younger rather than older women, (IARC, 2002; Ahn *et al*, 2004; Shen *et al*, 2005; Yang *et al*, 2005; Kuo *et al*, 2006). Because epidemiologic profiles and the availability and implementation of breast cancer screening and adjuvant systematic treatment programmes differ between Asian and Western countries, it is of great interest to assess whether incidence rates and prognoses for unilateral and bilateral breast cancers differ between these regions. Our previous studies in Taiwan showed that the incidence of bilateral breast cancer increased with time after the first primary breast cancer, and that the annual progression rate from unilateral to bilateral breast cancer was faster than that observed in Western countries (Kuo *et al*, 2006).

Regarding prognosis, previous studies comparing survival between Western women with bilateral *vs* unilateral breast cancer have yielded inconsistent results. Some studies found that bilateral breast cancer had a poorer prognosis than unilateral breast cancer (Alexander *et al*, 1989; Brenner *et al*, 1993; Nomura *et al*, 1999), whereas others showed similar prognoses for both types (Fisher *et al*, 1984; Mose *et al*, 1997; Newman *et al*, 2001). A recent large cohort study in Sweden reported a trend of declining incidence for synchronous bilateral cancer, similar to unilateral breast cancer, but an increasing trend for metachronous bilateral cancer (Hartman *et al*, 2007). The authors also found that metachronous bilateral cancer showed the highest mortality rate, followed by synchronous bilateral cancer and then unilateral breast cancer. (Hartman *et al*, 2007).

In Western studies, comparisons between unilateral and bilateral breast cancers are frequently based on cases diagnosed at greater than 50 years, of age; for this reason, relatively little is known regarding survival profiles for early-onset unilateral and bilateral breast cancers, which are frequently observed in modernising Asian countries. By following up with a previously described cohort of Taiwanese women with unilateral and bilateral breast cancers (which showed a preponderance of young women; Kuo *et al*, 2006), this study offers an opportunity to compare cumulative survival rates between unilateral and bilateral breast cancers while controlling for other prognostic factors associated with the risk of death using a time-dependent Cox regression model.

## Patients and methods

### Study subjects

A total of 1907 Taiwanese women with breast cancer, including 1863 unilateral and 44 bilateral cases of breast cancer, were recruited from among the patients treated at the National Taiwan University Hospital. All patients had been diagnosed with primary breast cancer between 1990 and 1999. Patients with bilateral breast cancer were further divided into two groups, synchronous and metachronous, with an interval between the first and contralateral breast cancer of ⩽6 months and >6 months, respectively. The mean (median) time until diagnosis of metachronous breast cancer was 3.03 (2.80) years. Because we had no data on the date of one patient's second diagnosis, only 43 patients were considered when synchronous and metachronous bilateral breast cancers were analysed separately. Basic characteristics and clinicopathological details were collected using the procedure for bilateral breast cancer cases described in detail elsewhere (Kuo *et al*, 2006). The treatment guideline for breast cancer follows the guideline set up by the organisation, called Taiwan Cooperative Oncology Group, which has been supported by the National Health Research Institute. The guidelines for breast cancer include surgery, adjuvant therapy, and surveillance schedule for different staging of breast cancer (http://english.nhri.org.tw/inst_cancer/ca_TCOG.php).

### Study design and data collection

A retrospective cohort study was designed to follow these 1907 breast cancer cases until 31 December 2004. The main variable of interest was the type of breast cancer (unilateral, metachronous bilateral, or synchronous bilateral). The primary end point was death from breast cancer. The mean follow-up time was 7.8±(s.d.), 3.8 years for all patients, compared to 7.9±3.8 years, 7.0±5.0 years, and 6.6±3.7 years for unilateral, synchronous bilateral, and metachronous bilateral cases, respectively.

Regarding confounding variables, a research assistant retrospectively retrieved information from medical charts. Data on demographic features, anthropometric measurements, reproductive factors (e.g., number of pregnancies, deliveries, and abortions), personal history of cancer and breast disease, family history of breast cancer among first- or second-degree relatives, and previous surgery or use of hormones were collected. Information on tumour attributes and surgical findings were first reviewed by the research assistant and then confirmed by specialists.

### Statistical analysis

The Wilcoxon's rank sum test was used to test differences in mean values for continuous variables between two groups. The *χ*^2^ test was used to compare categorical variables between groups, and the Fisher's exact test was applied if a sparse number in a specific cell was encountered. Cumulative survival was plotted for unilateral and bilateral (synchronous or metachronous) cancers using the Kaplan−Meier method. The log-rank test was used to determine whether differences among types of breast cancer were statistically significant. We used the indicator method to deal with covariates with missing values in the Cox regression model (Greenland and Finkle, 1995).

**Figure 1:**
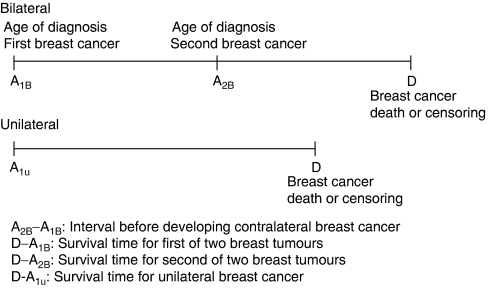
Time-dependent analysis of unilateral and bilateral breast cancer.

Figure 1 illustrates why a time-dependent Cox regression model is required to compare cumulative survival between unilateral and bilateral breast cancer. Women with bilateral breast cancer had two dates of diagnosis, one for the primary tumour (A_1B_) and one for the contralateral tumour (A_2B_); the duration between A_1B_ and A_2B_ is the interval between the first and second breast tumours, called waiting period for the development of the second cancer of bilateral type. D refers to breast cancer death, loss to follow-up, or the end of study. Thus, two survival intervals were defined for women with bilateral breast cancer (D−A_1B_ and D−A_2B_), but only one (D−A_1U_) for women with unilateral breast cancer. Because the comparison between unilateral (code=0) and bilateral breast cancer (code=1) should be made on the basis of D−A_1B_, the status (code=0) regarding the waiting period between the first of two breast tumours and the second (A_2B_–A_1B_) should be treated as if they are unilateral. The status (code=1) is then altered to bilateral breast cancer once the second is developed. Note that all tumour attributes corresponding to the first and second tumours and adjuvant treatments indicated by the physician during the first and second follow-up periods were treated as time-dependent covariates. The association between each relevant variable and the risk of death was assessed through univariate analysis. All significant variables in the univariate analysis were entered into the multivariate analysis. To optimise statistical efficiency, a parsimonious model was created using only variables retaining statistical significance after adjustment for all other relevant variables. Crude and adjusted hazard ratios (HRs) and their 95% confidence intervals were computed in univariate and multivariate analyses. The level of statistical significance was set at 5%.

## Results

Table 1 summarises demographic characteristics and reproductive and lifestyle factors for the study cohort. The mean ages at diagnosis in women with bilateral and unilateral breast cancers were 48.7±10.0 and 49.8±12.2 years, respectively; women with metachronous bilateral cancer showed the youngest age at onset (48.5±11.2 years). With the exception of family history, the remaining variables were not significantly associated with type of breast cancer. Women with a family history of breast cancer were more likely to develop contralateral breast cancer than women without.

**Figure 2:**
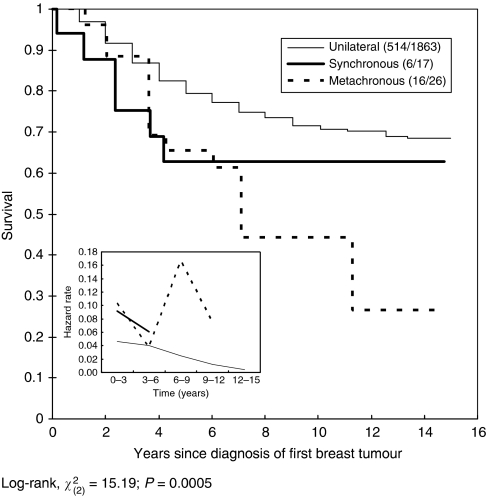
Cumulative breast cancer-specific survival according to type of breast cancer (unilateral *vs* bilateral).

**Figure 3:**
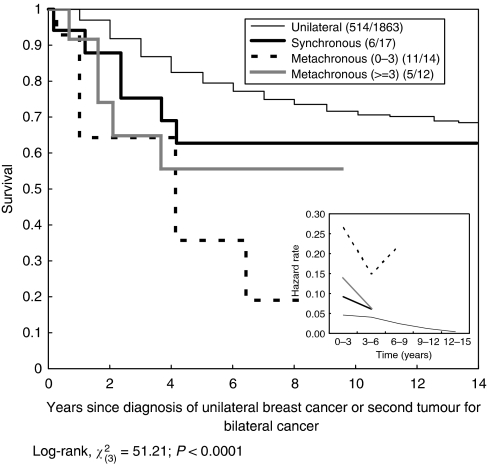
Cumulative breast cancer-specific survival rates for unilateral, synchronous bilateral, and metachronous bilateral breast cancers.

After 15 years of follow-up, the case fatality rate was lower in unilateral breast cancer (27.6%, 514 out of 1863) compared to synchronous bilateral breast cancer (35.3%, 6 out of 17) and metachronous bilateral breast cancer (61.5%, 16 out of 26). Figure 2 shows cumulative survival rates according to breast cancer type. Metachronous breast cancer was associated with the poorest survival rate, followed by synchronous breast cancer, compared to unilateral breast cancer. The 15-year survival rates were 68.4, 62.6, and 26.4% for unilateral, synchronous bilateral, and metachronous bilateral breast cancer, respectively, and this difference was statistically significant (*P*<0.01). However, the disparity between unilateral and metachronous bilateral cancer was larger than that between unilateral and synchronous bilateral breast cancer. The difference was most apparent after 5 years of follow-up. Interestingly, remarkably divergent time points occurred at approximately 3 years for unilateral and synchronous bilateral breast cancer and at 6 years for metachronous and synchronous bilateral breast cancer, implying that the average interval for developing contralateral breast cancer was approximately 3 years. Figure 3 shows cumulative survival for unilateral, synchronous, and metachronous cancer, as well as the interval between the diagnosis of the first and second tumours in women with metachronous bilateral breast cancer. Women in whom the second metachronous tumour was diagnosed within 3 years showed the poorest survival. It is also noted that Figures 2 and 3 show how hazard rates changed with time by types of breast cancer. The patterns of trend by type of breast cancer were consistent with the survival profiles.

The frequencies of clinical attributes according to type of breast cancer are listed in Table 2. Bilateral breast cancer patients were less immunoreactive for the oestrogen receptor than were unilateral breast cancer patients (*P*=0.04). Oestrogen receptor negativity was most prominent in patients with synchronous bilateral breast cancer (*P*=0.03). Synchronous breast cancer also showed a higher likelihood of being negative for the progesterone receptor (*P*=0.08). In bilateral breast cancer, metachronous cases had a higher likelihood of axillary lymph node involvement compared to synchronous cases (*P*=0.02). The likelihood of local recurrence (*P*=0.02) was higher in bilateral than in unilateral cases. Patients with metachronous bilateral breast cancer were more likely to have local recurrence (*P*=0.03) and distant metastasis (*P*=0.01) than those with synchronous cancer.

Table 3 shows the crude and adjusted HRs for relevant variables. Women with bilateral breast cancer had a 3.27-fold (95% CI, 2.15−4.97) greater risk of death compared to those with unilateral breast cancer. In the multivariate analysis, only the type of breast cancer, histological type, chemotherapy (only adjusted in the model analysing synchronous and metachronous bilateral breast cancer separately), tumour size, and regional lymph node involvement remained statistically significant after adjusting for significant variables. After controlling for these four variables, the HRs for death due to overall bilateral, synchronous bilateral, and metachronous bilateral breast cancer were 2.50 (95% CI, 1.43−4.37), 1.12 (95% CI, 0.42−3.02), and 6.11 (95% CI, 3.14−11.89) compared to unilateral breast cancer.

## Discussion

### Prognosis for unilateral and bilateral breast cancer in Asian countries

Complete follow-up data on the prognoses of unilateral and bilateral breast cancer in modernising Asian countries is scanty. For this reason, we conducted a long-term, longitudinal, follow-up study to examine demographic data, conventional risk factors, tumour attributes, systematic adjuvant therapy, and family history in a cohort of Taiwanese women. Our study demonstrates that patients in Taiwan with bilateral breast cancer, particularly of the metachronous type, showed poorer survival. In Taiwan, breast cancer is frequently diagnosed before 50 years of age (Kuo *et al*, 2006) and Taiwanese women with bilateral tumours face a 6-fold greater risk of death compared to women with unilateral breast cancer, after controlling for lymph node status, tumour size, histological type, and positive family history. The corresponding risk increased to 8-fold (95% CI, 3.00–23.32, *P*<0.0001) after further adjustment for age at diagnosis, adjuvant therapy, and progesterone and oestrogen status, although none of these was statistically significant in the multivariate analysis. These results suggest that poor survival in bilateral breast cancer patients cannot entirely be accounted for by family history, lack of adjuvant therapy, unfavourable tumour attributes (i.e., tumour size, lymph node status, and histological type), late detection, or other factors. Poor survival in bilateral cancer patients independent of unfavourable tumour attributes due to late diagnosis was also reported by Kollias *et al* (2001); in this previous study, bilateral breast cancer showed a 1.67-fold greater risk of death after adjustment using the Nottingham Prognostic Index, which considers tumour size, histological grade, and lymph node status. It is thought that survival rates are affected by other factors, such as access to medical care and lifestyle factors. However, these factors are unlikely to have had a significant effect in our analysis because they were similar across groups (Table 2).

After ruling out the possibilities mentioned above, a difference in survival remained, making it difficult to explain why bilateral breast cancer is associated with poorer survival compared to unilateral breast cancer. This may be partially explained by genetic reason. In our study, women with bilateral breast cancer had higher proportion of having family history than those with unilateral breast cancer when family history is a significant predictor for poor prognosis. The genetic susceptibility may also account for why synchronous breast cancer showed better survival than metachronous breast cancer. Women with metachronous bilateral breast cancer showed earlier onset than women with unilateral or synchronous bilateral cancer, and an interval of less than 3 years between metachronous tumours was associated with poor survival. Thus, in a country where breast cancer is frequently diagnosed in women of less than 50 years of age, poor survival may be attributed to metachronous bilateral cancers. Based on our results, we recommend that the inter-examination interval for unilateral breast cancer should not exceed 18 months (assuming uniform distribution of developing contralateral breast cancer). However, to prove the possibility of genetic susceptibility in relation to any survival difference across type of breast cancer molecular genetic studies such as BRCA1 and BRCA2 in association with survival, although the results are inconsistent (Moller *et al*, 2007; Rennert *et al*, 2007; Tutt and Ashworth, 2008), are still required.

### Comparison with findings from Western nations

The prevalence of young breast cancer patients in Asian countries may indicate that these women show distinct clinical profiles regarding the prognoses of unilateral and bilateral breast cancer. A comparison between our study and that of Hartman *et al* (2007) showed that the 10-year survival for metachronous bilateral breast cancer patients was markedly poorer among Taiwanese women (40%) compared to Swedish women (60%), whereas the corresponding figures for unilateral and synchronous bilateral breast cancers were similar. Because Hartman *et al* (2007) found a higher mortality rate for metachronous bilateral breast cancer in women younger than 50 years old, it stands to reason that the relatively poor survival among patients with metachronous bilateral breast cancer in our study is the result of a disproportionately large number of young breast cancer patients in our cohort. One may also speculate whether such a difference of survival is related to local recurrence and the finding of ER status. In Table 2, we have demonstrated metachronous cancer is more likely to show local recurrence and the greater frequency of ER-positive among metachronous cancer patients. The latter finding is consistent with the results of poor prognosis among young women with ER-positive breast tumour in comparison to negative hormonal profiles (Aebi *et al*, 2000; Ahn *et al*, 2007; Anders *et al*, 2008).

### Strength of study

One of the major merits of our study is the application of a time-dependent survival model. The rationale for using time-dependent Cox regression model is two-fold. First, this method overcomes the problem of selective survival for those who have survived longer and developed a second tumour in comparison to those who had the potential to develop bilateral tumours but did not do so before dying or reaching the end of the study period. As illustrated in Figure 1, which shows survival time without considering the altered status, the interval D−A_1B_ for bilateral breast cancer is greater than D−A_1U_ for unilateral breast cancer. However, if a waiting period is taken into account, the survival time for bilateral breast cancer would be shorter than that for unilateral breast cancer. The HR for metachronous bilateral breast cancer compared to unilateral breast cancer decreased to 3.11 (95% CI, 1.60–6.04) when a time-dependent covariate for type of breast cancer was not used. The second advantage of using time-dependent Cox regression model is to account for time-dependent covariates. (i.e., tumour attributes, and adjuvant therapy for the first tumour of bilateral breast cancer may be different from the second adjuvant therapy (EBCTCG, 2005a, 2005b)). This justified for using time-dependent Cox regression model.

Questions may be raised regarding the relatively small sample size examined here; however, such small cohorts cannot be avoided in Asian countries where the baseline bilateral breast cancer incidence remains relatively low. In contrast, in Sweden, Hartman *et al* (2007) were able to analyse a relatively large cohort of 6,550 bilateral breast cancer cases. After controlling for other significant prognostic factors, our results based on multivariate time-dependent Cox regression model still show statistically significant results for the difference of survival across unilateral and bilateral breast cancer types, given a significant statistical level and 95% confidence interval not including 1 for adjusted hazard ratios. We do not think statistical power is a serious problem. Admittedly, however, our small sample may have created a greater degree of variation in our results. Consequently, further large-scale studies in Asian nations are required to validate our results.

### Implications for regions with low incidence but predominantly young onset

Despite a number of studies on the prognosis of unilateral and bilateral breast cancer in Western countries, our study may have significant implications for modernising Asian nations in which the incidence of unilateral breast cancer is high and post-menopausal women are predominantly affected. In developing Asian nations, women between the ages of 45 and 54 show the highest incidence of breast cancer; in contrast, women above the age of 65 are more commonly affected in the United States and the United Kingdom (IARC, 2002). The reasons for these distinct epidemiological patterns remain obscure, but three possibilities have been proposed. The first hypothesis is that older Asian women are less susceptible to breast cancer than young Asian women because of their relatively lower lifetime oestrogen exposure. That is, older Asian women are more likely to have experienced late menarche, earlier age at first full-term pregnancy, higher number of births, earlier menopause, and low utilisation of oral contraceptive pills or hormone replacement therapies compared to their younger counterparts, who have received oestrogen exposure similar to that experienced by women living in Western nations. Such a cohort-driven effect has been demonstrated in several modernising Asian studies (Seow *et al*, 1996; Minami *et al*, 2004; Chia *et al*, 2005; Shen *et al*, 2005).

The second hypothesis is that early age at diagnosis may indicate that young Asian women are more likely to be genetically predisposed to breast cancer. The higher risk for breast cancer in young women among the Jews has been found to be associated with genetic predisposition (FitzGerald *et al*, 1996; Rubinstein, 2004). However, this postulate has been refuted by migration studies examining second generation Japanese migrant women, who show breast cancer rates similar to those of Caucasian women, but different from those of native Japanese women (Ziegler *et al*, 1993; Matsuno *et al*, 2007). Environmental factors have also been implicated by a second migration study showing that American-born Asian-Americans experienced earlier menarche than Asian women who immigrated to the USA (Shen *et al*, 2005).

The third hypothesis is that the age distribution of breast cancer patients in Western countries may be affected by the more frequent use of mammography among older women compared to younger women. However, the vast majority of modernising Asian countries lack population-based mammography screening. This may partly account for why breast cancer clusters peak around 60–69 years in Western countries.

These biological features together with the implementation of screening and systematic adjuvant treatment programmes suggest that the prognoses of unilateral and bilateral breast cancers in modernising Asian countries are distinct from those observed in Western nations.

In conclusion, metachronous bilateral breast cancers, particularly those that develop within an interval of less than 3 years, have a poorer prognosis than synchronous bilateral and unilateral breast cancers among Taiwanese women even after adjustment for tumour size, nodal involvement, and histological type. Survival among Taiwanese metachronous bilateral breast cancer patients was also lower than survival among patients in Sweden. Our results emphasise the importance of identifying those with the potential to develop metachronous bilateral breast cancer within 3 years after developing unilateral breast cancer.

## Figures and Tables

**Table 1:** Distributions of demographic, lifestyle, and reproductive features of unilateral and bilateral breast cancer cases, at time of first diagnosis

			**Bilateral**							
	**Unilateral (*N*=1863)**		**Synchronous (*N*=17)**		**Metachronous (*N*=26)**		**Overall (*N*=43)**			
**Variable**	**No.**	**%**	**No.**	**%**	**No.**	**%**	**No.**	**%**	***P*-value^a^**	***P*-value^b^**
Age(years)^c^	49.8±12.2 (47.9)		49.0±8.1 (46.1)		48.5±11.2 (50.1)		48.7±10.0 (49.5)		0.85	0.95
BMI	23.4±3.6		22.8±4.1		22.4±2.9		22.6±3.4		0.34	0.83
*Education (%)*									0.68	0.48
College or above	382	27.3	4	33.3	3	21.4	7	26.9		
Senior high school	340	24.3	3	25.0	1	7.1	4	15.4		
Junior high school	181	12.9	1	8.3	4	28.6	5	19.2		
Elementary school	351	25.1	3	25.0	3	21.4	6	23.1		
Illiterate	144	10.3	1	8.3	3	21.4	4	15.4		
*Age at menarche*	14.4±1.7		14.1±1.6		14.6±1.8		14.4±1.7		0.91	0.29
*Menopause*									0.07	0.70
Yes	739	55.8	8	66.7	15	75.0	23	71.9		
No	585	44.2	4	33.3	5	25.0	9	28.1		
*Age of menopause*	48.4±5.3		49.3±4.9		48.3±6.4		48.7±5.9		0.69	0.52
*Hysterectomy*									0.78	1.00
Yes	146	8.4	1	5.9	3	11.5	4	9.3		
No	1585	91.6	16	94.1	23	88.5	39	90.7		
Pregnancy	3.3±2.2		2.5±1.3		3.0±2.2		2.8±1.8		0.26	0.49
Childbirth	2.6±1.7		2.1±1.2		2.4±1.6		2.2±1.4		0.41	0.48
Miscarriage	0.7±1.1		0.4±0.6		0.6±1.1		0.5±0.9		0.33	0.91
Age of first childbirth	25.5±4.6		23.5±3.9		25.9±5.6		25.0±5.0		0.41	0.34
*Breast feeding*									0.15	0.34
Yes	420	60.8	1	20.0	7	50.0	8	42.1		
No	271	39.2	4	80.0	7	50.0	11	57.9		
*Smoking*									1.00	1.00
Yes	47	2.6	0	0.0	1	4.2	1	2.6		
No	1739	97.4	15	100.0	23	95.8	38	97.4		
*Drinking*									0.65	0.14
Yes	65	3.6	2	13.3	0	0.0	2	5.1		
No	1722	96.4	13	86.7	24	100.0	37	94.9		
Family history of breast cancer									0.05	1.00
Yes	86	4.6	2	11.8	3	11.5	5	11.6		
No	1777	95.4	15	88.2	23	88.5	38	88.4		

a Unilateral *vs* Bilateral.

b Synchronous *vs* Metachronous.

c The medium age was reported in the bracket.

**Table 2:** Distributions of clinical attributes and adjuvant therapies used in unilateral and bilateral breast cancer cases, at the time of first diagnosis

			**Bilateral**							
	**Unilateral (*N*=1863)**		**Synchronous (*N*=17)**		**Metachronous (*N*=26)**		**Overall (*N*=43)**			
**Variable**	**No.**	**%**	**No.**	**%**	**No.**	**%**	**No.**	**%**	***P*-value^a^**	***P*-value^b^**
*Tumour size (cm)*	3.3±3.7		4.0±2.8		3.5±2.0		3.7±2.3		0.33	0.48
T1 (⩽2)									0.48	0.67
T1a (⩽0.5)	16	0.9	0	0.0	0	0.0	0	0.0		
T1b (0.5−1.0)	100	5.9	2	13.3	1	5.0	3	8.6		
T1c (1.0−2.0)	464	27.2	3	20.0	6	30.0	9	25.7		
T2 (2.0−5.0)	922	54.1	6	40.0	10	50.0	16	45.7		
T3, T4 (>5)	201	11.8	4	26.7	3	15.0	7	20.0		
										
*Histology type*									0.18	0.13
IDC	1558	85.4	12	80.0	25	96.2	37	90.2		
DCIS	106	5.8	2	13.3	1	3.8	3	7.3		
ILC	30	1.6	1	6.7	0	0.0	1	2.4		
Others	130	7.1	0	0.0	0	0.0	0	0.0		
										
*Grade*									0.72	0.68
1	126	29.7	2	66.7	1	20.0	3	37.5		
2	206	48.6	1	33.3	2	40.0	3	37.5		
3	92	21.7	0	0.0	2	40.0	2	25.0		
										
*Oestrogen receptor*									0.04	0.03
Positive	871	64.2	4	26.7	12	63.2	16	47.1		
Negative	485	35.8	11	73.3	7	36.8	18	52.9		
										
*Progesterone receptor*									0.23	0.08
Positive	788	60.2	5	33.3	12	63.2	17	50.0		
Negative	521	39.8	10	66.7	7	36.8	17	50.0		
										
*Chemotherapy*									0.13	0.74
Yes	1080	59.8	12	75.0	18	69.2	30	71.4		
No	725	40.2	4	25.0	8	30.8	12	28.6		
										
*Radiotherapy*									0.12	1.00
Yes	164	10.0	3	18.8	4	16.7	7	17.5		
No	1480	90.0	13	81.2	20	83.3	33	82.5		
										
*Tamoxifen*									0.31	0.71
Yes	1444	81.4	12	80.0	18	72.0	30	75.0		
No	331	18.6	3	20.0	7	28.0	10	25.0		
										
*Axillary lymph node metastasis*									0.49	0.02
Yes	599	44.3	4	26.7	15	65.2	19	50.0		
No	753	55.7	11	73.3	8	34.8	19	50.0		
										
*Local recurrence*									0.02	0.03
Yes	203	11.2	1	5.9	9	36.0	10	23.8		
No	1607	88.8	16	94.1	16	64.0	32	76.2		
										
*Distant metastasis*									1.00	0.01
Yes	371	20.5	0	0	8	32.0	8	19.0		
No	1439	79.5	17	100	17	68.0	34	81.0		
										
*Metastatic sites*										
Bone	187	11.2	0	0.0	3	13.6	3	7.7	0.80	0.24
Lung	181	10.9	0	0.0	4	17.4	4	10.0	1.00	0.12
Liver	169	10.2	0	0.0	0	0.0	0	0.0	0.045	—
Brain	64	4.1	0	0.0	0	0.0	0	0.0	0.50	—
Neck LN	63	4.0	0	0.0	4	17.4	4	10.0	0.08	0.12

DCIS=ductal carcinoma *in situ*; IDC=infiltrating ductal carcinoma; ILC=infiltrating lobular carcinoma.

a Unilateral *vs* Bilateral.

b Synchronous *vs* Metachronous.

**Table 3:** Crude and adjusted hazard ratios (HRs) and 95% confidence intervals (CIs) for prognostic factors associated with the risk of breast cancer death

	**Crude HR**			**Adjusted HR (CR)^a^**			**Adjusted HR (CR)^b^**		
			***P*-value**			***P*-value**			***P*-value**
*Age at first onset (years)*						0.1927			
<35	1.23	(0.93−1.62)	0.15	1.20	(0.78−1.84)				
35−44	0.94	(0.77−1.13)	0.50	0.81	(0.60−1.09)				
⩾45	1.00	—		1.00	—				
									
*Type*									
Unilateral	1.00	—		1.00	—		1.00	—	
Bilateral	3.27	(2.15−4.97)	<0.0001	3.04	(1.97−4.69)	<0.0001	2.50	(1.43−4.37)	0.001
*Type 2*						0.0002			<0.0001
Unilateral	1.00	—		1.00	—		1.00	—	
Synchronous	1.52	(0.68−3.40)	0.31	2.91	(0.40−21.46)		1.12	(0.42−3.02)	
Metachronous	5.68	(3.45−9.37)	<0.0001	8.35	(2.99−23.32)		6.11	(3.14−11.89)	
									
*Histology type*						0.0013			0.0206
IDC	0.98	(0.73−1.30)	0.88	1.00	(0.74−1.35)		0.92	(0.69−1.24)	
DCIS	0.23	(0.12−0.47)	0.23	0.27	(0.13−0.54)		0.34	(0.17−0.69)	
ILC	1.30	(0.68−2.49)	0.43	1.16	(0.60−2.23)		1.15	(0.60−2.22)	
Others	1.00	—		1.00	—		1.00	—	
									
*Oestrogen receptor*									
−	1.00	—		1.00	—				
+	0.74	(0.61−0.91)	0.004	0.80	(0.59−1.08)	0.15			
									
*Progesterone receptor*									
−	1.00	—		1.00	—				
+	0.78	(0.64−0.96)	0.02	0.77	(0.57−1.03)	0.08			
									
*Chemotherapy*									
−	1.00	—		1.00	—				
+	3.36	(2.70−4.20)	<0.0001	1.40	(0.98−1.98)	0.06			
									
*Radiotherapy*									
−	1.00	—		1.00	—				
+	1.49	(1.14−1.95)	0.004	1.11	(0.73−1.70)	0.62			
									
*Tamoxifen*									
−	1.00	—		1.00	—				
+	1.28	(1.00−1.63)	0.047	1.33	(0.93−1.89)	0.12			
									
*Axillary lymph node metastasis*									
−	1.00	—		1.00	—		1.00	—	
+	4.10	(3.24−5.18)	<0.0001	2.56	(1.84−3.56)	<0.0001	3.43	(2.67−4.42)	<0.0001
									
*Size (cm)*									
<2	1.00	—		1.00	—		1.00	—	
⩾2	2.50	(1.84−3.38)	<0.0001	1.59	(1.06−2.39)	0.02	1.85	(1.30−2.62)	0.0006
									
*Family history*									
−	1.00	—		1.00	—		1.00	—	
+	1.44	(1.02−2.03)	0.04	1.42	(1.01−2.01)	0.04	1.49	(1.06−2.11)	0.02

DCIS=ductal carcinoma *in situ*; IDC=infiltrating ductal carcinoma; ILC=infiltrating lobular carcinoma.

a Controlling for all variables.

b Controlling for significant variables in multivariate analysis, including type of breast cancer, histology type of breast cancer, tumour size, regional lymph node involvement, and family history when overall bilateral breast cancer was compared with unilateral breast cancer; chemotherapy was also considered when metachronous and synchronous breast cancer were compared with unilateral breast cancer. Note that the adjusted HRs for the controlling variables provided in Table 3 were based on the model comparing overall bilateral breast cancer and unilateral breast cancer.
